# Monitoring of the Pesticide Droplet Deposition with a Novel Capacitance Sensor

**DOI:** 10.3390/s19030537

**Published:** 2019-01-28

**Authors:** Pei Wang, Wei Yu, Mingxiong Ou, Chen Gong, Weidong Jia

**Affiliations:** 1 Key Laboratory of Modern Agricultural Equipment and Technology, Ministry of Education of PRC, Jiangsu University, Zhenjiang 212300, China; myomx@ujs.edu.cn (M.O.); chengong@ujs.edu.cn (C.G.); 2 Key Laboratory of Plant Protection Engineering, Ministry of Agriculture and Rural Affairs of PRC, Jiangsu University, Zhenjiang 212300, China; 2211616028@stmail.ujs.edu.cn

**Keywords:** capacitor sensor, deposit mass, pesticide droplets, formulations, ionization

## Abstract

Rapid detection of spraying deposit can contribute to the precision application of plant protection products. In this study, a novel capacitor sensor system was implemented for measuring the spray deposit immediately after herbicide application. Herbicides with different formulations and nozzles in different mode types were included to test the impact on the capacitance of this system. The results showed that there was a linear relationship between the deposit mass and the digital voltage signals of the capacitance on the sensor surface with spray droplets. The linear models were similar for water and the spray mixtures with non-ionized herbicides usually in formulations of emulsifiable concentrates and suspension concentrates. However, the ionized herbicides in formulation of aqueous solutions presented a unique linear model. With this novel sensor, it is possible to monitor the deposit mass in real-time shortly after the pesticide application. This will contribute to the precision application of plant protection chemicals in the fields.

## 1. Introduction

The usage of pesticides has increased significantly in the last two decades in China [1]. Meanwhile, its average application dose is 2.5 times of the global average level [2,3]. The over usage of pesticides will cause unnecessary invest for the farmers, leading to more residue of the pesticide ingredients in the food products and the soil, and damage the eco-system as well [4,5,6]. Furthermore, it can also induce the resistant property of the weeds, insects, and diseases, which makes the pest management strategies more complicated [7,8]. Thus, the effective approaches to monitor the droplet deposition doses during the pesticide application are in urgent demands.

To enhance the assessment efficiency of the deposition quality for the spray, several approaches have been introduced based on the image processing technology [9]. Various systems have been open for users to identify the pesticide deposition effect by analyzing the images of the water sensitive paper after the application, for instance, the Swath Kit [10], the USDA Image Analyzer [11], the Droplet Scan [12], the Deposit Scan [13], the Image J [14], and the Drop Vision-Ag [15]. Most of the new technologies are applied for measuring the coverage, droplet density, and or the droplet sizes. Few methodologies have been reported to evaluate the droplet deposition doses after pesticide application.

Conventionally, the droplet deposition dose is measured based on the elution procedure [16]. Pigments like the poinsettia and the methylene blue are usually selected to replace the pesticide for the spray preparation. After the application, plant leaves or the Petri dishes will be collected and the pigment ingredients will be eluted with deionized water from the leaf surfaces or the Petri dishes. Then the deposition dose can be calculated from the concentration of the eluent by the measurement with a spectrophotometer [17]. Salyani and Serdynski [18] reported a prototype sensor for the spray deposit monitoring based on the measurement of the electric capacitance of the parallel copper conductors with spray deposited between the copper gaps. Thus, it could indicate the mass information of the droplet deposit with the voltage signals, which enables the real-time and rapid measurements of the pesticide deposition doses. Recently, Zhang et al. [19] applied a similar system for droplet deposition evaluation of the aerial spraying. Meanwhile, a fringing capacitive sensor with similar principles was also applied for water content measurement in the field [20]. However, the prototype equipment was tested with the electrolyte solution with high conductivity, such as NaCl and NaOH, which is not a common feature of the typical spray mixture. As is known, besides the water solvent and the emulsion in water with the ingredients as ionic compound, most pesticide formulations with the organic ingredients are hardly ionized in the water mixture. Thus, further studies are required to verify the fitness and the measurement accuracy of this novel sensor before its application in the agricultural practice.

The objectives of this study are, firstly, to test the sensors application capability on the deposition dose measurement of spray mixtures with pesticide in different formulations; secondly, to evaluate the measuring accuracy of droplet deposition doses of spray mixture with several common pesticide formulations including the emulsifiable concentrates (EC), suspension concentrates (SC), and aqueous solutions (AS).

## 2. Materials and Methods

### 2.1. Implementation of the Droplet Deposit Sensing System

**Figure 1:**
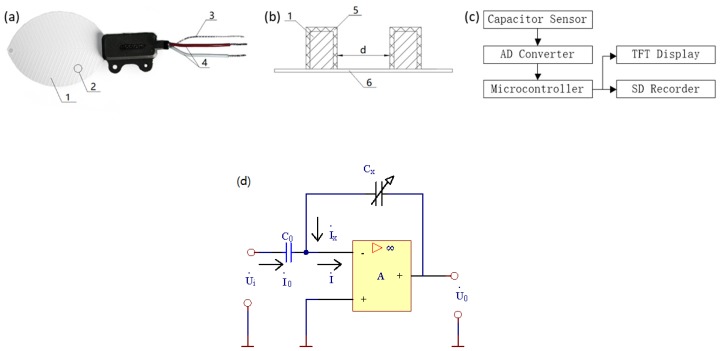
The structure of the leaf-like capacitor sensor and the deposit monitoring system. (**a**) Is the leaf-like sensor. (**b**) Is the electrode structure on the resin board. (**c**) Is the implementation diagram of the deposit monitoring system. (**d**) Is an electric schematic of the circuit of the leaf like sensor. 1 = electrodes, 2 = capacitor, 3 = data cable, 4 = power cable, 5 = insulating coating, 6 = resin board. The sensor is in 11.2 cm of length, 5.8 cm of width and 0.075 cm of thickness. The distance between each electrode is 1.58–1.78 mm. The width of the electrode is 0.59–0.79 mm and the thickness is 0.01–0.02 mm. The thickness of the insulating layer is about 2 μm. In (**d**), *C_0_* is a fixed capacitor, *C_x_* is the leaf like sensor.

A leaf like capacitor (Yingtai Tech., Tianjin, China) was adopted for the implementation of the droplet deposit sensing system in this study. The sensor was designed based on a capacitor with 84 parallel coppers (Figure 1). The coppers were separated into two groups and connected respectively as two electrode plates of the capacitor. The whole structure of the capacitor was packaged (painted) with insulation material of ceramic. Lim et al. has presented that, the capacitance varies according to the dielectric constant of the media composition, the air or the spray, inside the gap of the electrodes [21]. The dielectric constant changes when the ratio of each component in the media composition varies. The dielectric constant can be calculated due to the equation

(1) $$C_{s}=\frac{\varepsilon_{a}S_{a}+\varepsilon_{s}S_{s}}{d}$$

where *C_s_* is the capacitance of the capacitor with droplets depositing onside, *ε_a_* is the dielectric constant of the air, *ε_s_* is the dielectric constant of the spray mixture, *S_a_* is the surface area of the electrode contacting to the air, *S_s_* is the surface area of the electrode contacting to the spray droplet, and d is the distance between the copper electrodes.

When considering the total area of the electrodes (*S*), which should be the sum of *S_a_* and *S_S_*, the equation of the dielectric constant can be revised as

(2) $$C_{s}=\frac{\varepsilon_{a}\left(S-S_{s}\right)+\varepsilon_{s}S_{s}}{d}=\frac{\varepsilon_{a}S}{d}+\frac{\left(\varepsilon_{s}-\varepsilon_{a}\right)S_{s}}{d}=C_{0}+\frac{\left(\varepsilon_{s}-\varepsilon_{a}\right)S_{s}}{d}$$

where, *C*_0_ is a constant for each sensor.

Theoretically, the surface area of the electrode contacting to the spray droplet (*S_S_*) can be calculated from the droplets’ mass (*m_s_*) and density (*ρ_s_*). Then, the deposit mass of the spray can be calculated following the equations

(3) $$m=\rho_{s}\;S_{s}d=\rho_{s}\frac{\left(C_{s\;}-C_{0\;}\right)d}{\;\varepsilon_{s}-\;\varepsilon_{a}}d=C_{s}\frac{\rho_{s}d^{2}}{\;\varepsilon_{s}-\;\varepsilon_{a}}-\frac{\rho_{s}C_{0\;}d^{2}}{\;\varepsilon_{s}-\;\varepsilon_{a}}$$

Thus, a linear model can be fitted to calculate the deposit mass of the spray according to the measurement of the capacitance of the capacitor with droplets depositing onside.

With the leaf like capacitor adopted in this study, it could provide the analog signals to indicate its capacitance. The sensor is implemented with an operational amplifier circuit as shown in Figure 1d. With a capacitor in fixed capacitance, the leaf like capacitance sensor will share different voltages when the dielectric constant inside it is changed. The capacitor was linked to an analog to digital converter (ADS1115, Texas Instruments, Dallas, TX, USA), which could convert the analog signal of the real time capacitance into the digital voltage signals. The digital signal was then processed by a 32-bit microcontroller (STM32F4 EXPLORER, ST Microelectronics, Geneva, Switzerland). The microcontroller could read the input digital signal and display the voltage information representing the analog signal on a thin film transistor-liquid crystal display (TFT screen). Linear models of deposit mass to the voltage signals were developed due to different herbicide spray in this research and installed in the microcontroller. Thus, the microcontroller could calculate the deposit results of each measurement and then display that on the TFT screen. All the measurement results were recorded with a trans-flash Card (SD card) which was mounted on the microcontroller processing board. Figure 1 presents the structure of the system.

### 2.2. Experimental Setup and Processing

**Figure 2:**
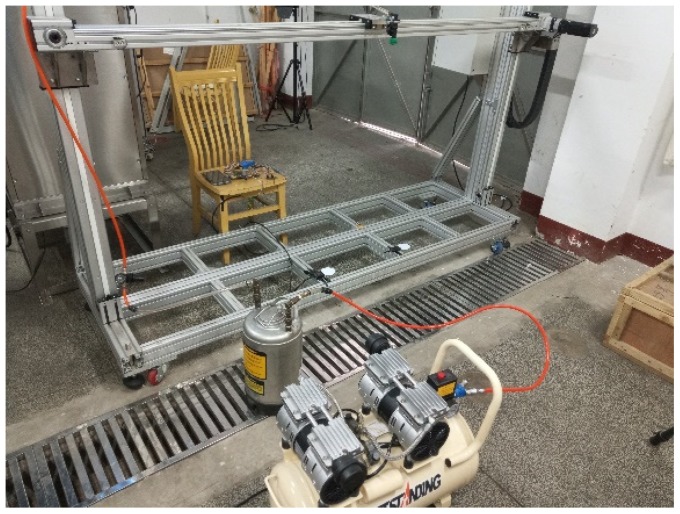
The hanging orbit sprayer for herbicide application in the experiment.

To develop the models of deposit mass to the voltage signals, experiments were conducted to measure the voltage signals of the leaf like sensor with different herbicide spray depositing onside. Herbicides in different formulation types were selected for the spray preparation with the suggested concentration on the product labels including the Yudasheng^®^ (SC, 20% a.i. atrazine, Xinnong Guotai, Beijing, China), Lvlilai Butachlor^®^ (EC, 50% a.i. butachlor, Lvlilai, Suzhou, China), Ruidefeng Lanhuoyan^®^ (AS, 41% a.i. glyphosate isopropylamine salt, Noposion, Shenzhen, China), as well as the water for control. The sprays were prepared according to the details listed in Table 1. A hanging orbit sprayer (Figure 2) was used for the herbicide application. The application pressure was set at 0.3 MPa, while the application height was set at 50 cm above the sensor as is common in field conditions. Standard flat fan nozzles with different sizes were selected for the spraying (Lechler^®^ ST 110-01/015/02/03, Lechler GmbH, Metzingen, Germany). Before spraying onto the sensors, the medium droplet sizes were measured for each nozzle with a laser particle size analyzer (Winner^®^ 318B, Winner Particle Instrument, Ji’nan, China). During the testing experiment of the sensor, the moving velocity of the nozzles was fixed for three repeated measurements of each treatment and then varied randomly to obtain different deposit on the sensor surface. For each application, the signals from the sensor were recorded for 40 times over three seconds after the application. Mass of the sensor was measured with an electrical scale (YP102N, Sop-Top, Shanghai, China) before and after application. The deposit mass was calculated from the two mass measurements. 

### 2.3. Data Analysis

The data analysis was processed with R Studio 0.98.490 [22]. The linear model was employed to fit the relationship between the voltage signals and the deposit mass of the spray. Analysis of variance (ANOVA) was carried out for the evaluation of differences between each treatment. Data were tested for normal distribution using the Shapiro-Wilk test (*p* > 0.05). Equality for heterogeneity of variances was tested using Levene’s test for each treatment (*p* > 0.05).

## 3. Results and Discussion

### 3.1. Measurement Impact Analysis

**Figure 3:**
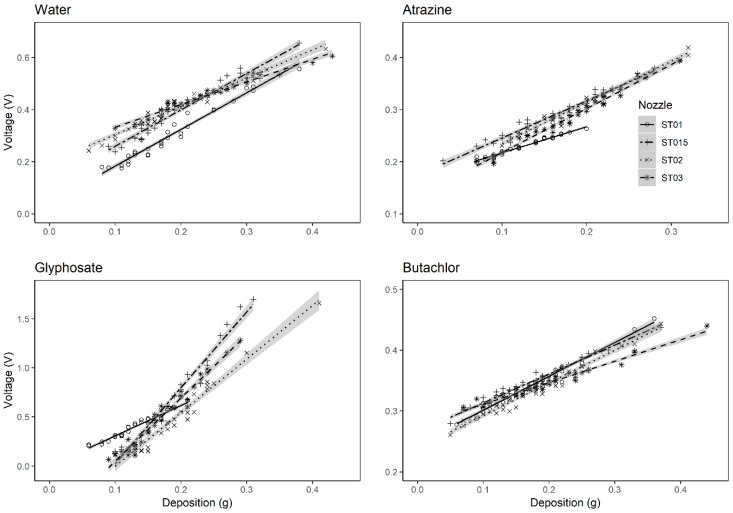
Linear regression models of the deposit data of sprays with herbicides in different formulations. The shadow area represents the standard error of the regressed linear models.

**Figure 4:**
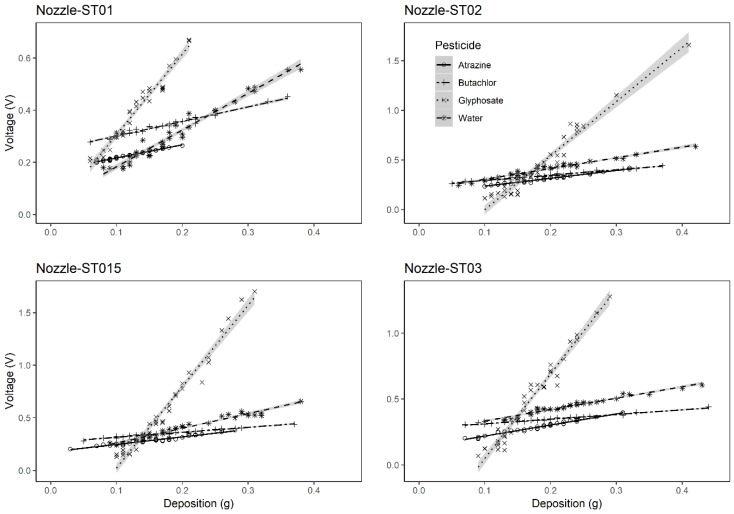
Linear regression models of the deposit data of sprays with droplet size spectrum generated from different nozzles. The shadow area represents the standard error of the regressed linear models.

To evaluate the impact of application factors as the nozzle types (droplet sizes) and the formulation types of the herbicides on the sensor’s measurement results, experiments were conducted. The measuring results were presented in Figure 3 and Figure 4.

Linear models were employed for the regression of the signal voltage to deposit mass curves of each treatment. All the models were compared by ANOVA (α = 0.05). It indicated that, when the nozzle type was fixed, there was no significant differences between the deposit to voltage curves of the spray mixture with EC (butachlor), SC (atrazine), and water. However, considering the AS (glyphosate), the voltage signal curve of the spray deposit was markedly different from the other groups. When the deposit is greater than 0.2 g on the leaf like sensor, the signal voltages were much higher of the sensor with AS droplets than the EC, SC, or water droplets. To explain this difference, the ionization property of the glyphosate isopropylamine salt should be concerned. The glyphosate is a weak acid herbicide. It usually exists as a salt compound and will divide into two ions with opposite charges (the cation and the anion) after being dissolved in the water, while the other herbicides with formulations like EC or SC usually contain organic ingredients which could not dissolve and ionize in the water. The concentration of ions would vary the dielectric constant of the spray mixture. As a result, the voltage signals of the electric capacity would be higher when the concentration of the glyphosate was increased. 

In Figure 4, deposit curves of different nozzles were compared on each herbicide formulation. It presented that, for the spray mixture with same herbicide formulations, there was no significant differences of the deposit curves between the treatments with each nozzle type. Therefore, the spray mixture could be classified into two groups due to the ionization feature of the ingredients.

### 3.2. Statistical Modeling

Considering the little differences of the deposit curves of water, SC and EC sprays, the data could be gathered for the modeling of the signal voltage to the deposit mass for all of the non-ionized herbicides, while the ionized herbicides should be tested for separated models. In this research, the glyphosate was taken as an example of the cases for ionized ingredients application. Since, the nozzle types did not show any impact on the signal voltages of the sensor measurement, the data of different herbicides but with the same nozzle type could be gathered for the modeling. Linear models were applied respectively for the regression of the two cases. The linear regression fitted well to the grouped data, which corresponding to the results of a former study by Zhang et al. [19]. 

**Figure 5:**
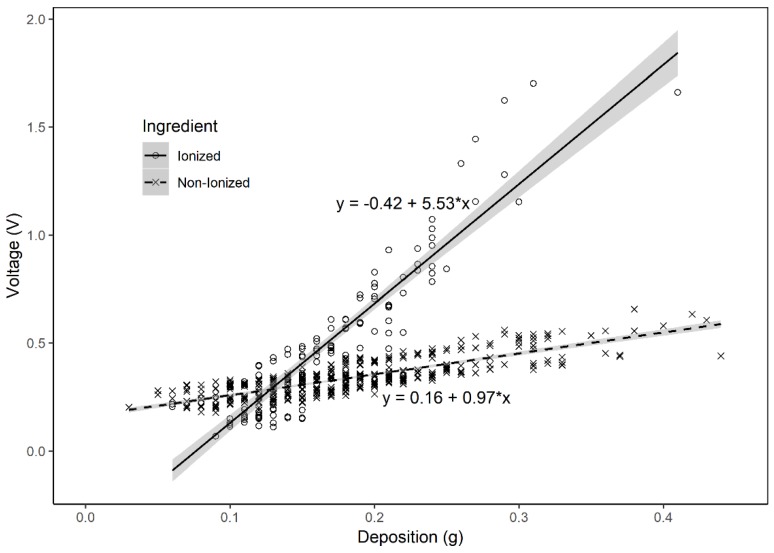
Linear regression models of the deposit data of sprays with ionized and non-ionized ingredients. The shadow area represents the standard error of the regressed linear models (ionized: R^2^ = 0.837, non-ionized: R^2^ = 0.882).

With the linear model of the electric signals to the deposit of the non-ionized sprays, the algorithm could be programmed and installed in the microcontroller. Thus, the sensor was able to give precise information of the quantified spray deposit in the field. The dielectric constant of a dielectric material depends on the frequency of the applied electric field. The behavior of the dielectric constant is affected by three types of polarization including the orientation polarization, the ionic polarization and the electronic polarization. The ionized sprays may show all the three types of polarization, so that all polarization mechanisms are acting at lower frequencies till the water dipoles fail to follow the field alternations and so the orientation polar ceases to play, after which the polarization is dominated by ionic polarization. This continues until the ions’ oscillations cannot follow the field alternation and it gets out the play at which the reaming will be the electronic polarization. When the frequency of the applied field equal to the resonance frequency of ionic movement, the dielectric constant show resonance behavior. The dielectric constant is proportional to density of the induced dipoles. Therefore, it is expected to increase with the salt concentration of the electrolyte [23]. Thus, the electric signal of ionized spray will be affected by the mount, concentration and other parameters of the electric charges in the mixture. Therefore, the model of one variable regression could not be used to simulate the application deposit with different ingredients. However, in this certain case of glyphosate application with the recommended doses on the product label, the linear regression model presented in Figure 5 could be adopted with the algorithm program similar to the one used for the deposit detection of the non-ionized sprays.

## 4. Conclusions

According to this study, we can conclude that the electric capacitor sensor has shown strong potential for its application on the real-time monitoring of the herbicide spraying deposit. The system and model implemented in this study can already be applied in the deposit measurement of the herbicide with non-ionized ingredients. For other water-soluble herbicides with ionized ingredients. However, further studies are required for the measuring model simulation, as the concentration and the valence of the ions will have impact on the dielectric constant of the spray mixture, which can directly affect the signal voltage representing the electric capacity of the sensor.

## Figures and Tables

**Table 1:** Preparation of the sprays of the experiment

No	Formulation	Ingredient	Product Amount	Solution/Water Volume (mL)
1		water		5000
2	SC	atrazine	75 mL	5000
3	EC	butachlor	50 g	5000
4	AS	glyphosate isopropylamine salt	225 mL	5000
