# Erratum: Wang, P., et al. Monitoring of the Pesticide Droplet Deposition with a Novel Capacitance Sensor. *Sensors* 2019, *19*, 537

**DOI:** 10.3390/s19051022

**Published:** 2019-02-28

**Authors:** Pei Wang, Wei Yu, Mingxiong Ou, Chen Gong, Weidong Jia

**Affiliations:** 1 Key Laboratory of Modern Agricultural Equipment and Technology, Ministry of Education of PRC, Jiangsu University, Zhenjiang 212300, China; myomx@ujs.edu.cn (M.O.); chengong@ujs.edu.cn (C.G.); 2 Key Laboratory of Plant Protection Engineering, Ministry of Agriculture and Rural Affairs of PRC, Jiangsu University, Zhenjiang 212300, China; 2211616028@stmail.ujs.edu.cn

The authors wish to make the following correction to this paper [1]:

The first two authors Pei Wang and Wei Yu contributed equally to this publication as the co-first authors. As the first authors, their roles and authorship order in this publication should both be regarded as equal.

The authors would like to apologize for any inconvenience caused to the readers by this change.
